# Early Chronic Kidney Disease (G1-G3a) in Combination with Steatosis as a Predictor of Incident Ischemic Heart Disease: A Longitudinal Study in Non-Diabetic Koreans

**DOI:** 10.3390/biomedicines9101358

**Published:** 2021-09-29

**Authors:** Sung-Bum Lee, Byoung-Jin Park, Yong-Jae Lee, Dong-Hyuk Jung

**Affiliations:** 1 Department of Health Check-up, Yongin Severance Hospital, Yongin-si 16995, Korea; DOLSOUI@yuhs.ac; 2 Department of Family Medicine, Yongin Severance Hospital, Yongin-si 16995, Korea; bjpark96@yuhs.ac; 3 Department of Family Medicine, Gangnam Severance Hospital, Seoul 06273, Korea; ukyjhome@yuhs.ac

**Keywords:** chronic kidney disease, hepatic steatosis, longitudinal study, ischemic heart disease

## Abstract

Hepatic steatosis and chronic kidney disease (CKD) in the advanced stages are closely related to cardiovascular diseases. Despite the potential connection between early CKD (G1-G3a) and hepatic steatosis on cardiometabolic risks, few studies have revealed their causal link to ischemic heart disease (IHD). We prospectively investigated the combined effect of CKD in earlier stages and hepatic steatosis on incident IHD risk in large-scale, non-diabetic Koreans. Data were assessed from 16,531 participants without diabetes from the Health Risk Assessment Study (HERAS) and Korea Health Insurance Review and Assessment (HIRA) data. We divided the study population into four groups according to the existence of early CKD and hepatic steatosis: controls, early CKD only, hepatic steatosis only, and both early CKD and hepatic steatosis. We prospectively assessed hazard ratios (HRs) with 95% confidence intervals (CIs) for IHD using multivariate Cox proportional-hazard regression models over a 50-month period. During the follow-up period, 326 (2.0%) patients developed IHD. HRs of IHD in the four groups were 1.00 (controls), 1.26 (95% CI 0.72–2.19), 1.19 (95% CI 0.90–1.57) and 1.76 (95% CI 1.04–2.97), respectively, after adjusting for potential confounding variables. Even less than stage 3A, CKD could precede and predict IHD in patients with hepatic steatosis.

## 1. Introduction

Both chronic kidney disease (CKD) and hepatic steatosis have been increasing over the decades; simultaneously, the healthcare burden is gradually increasing for managing each of these diseases. The mean global prevalence of CKD is 13.4% in all stages [1]. CKD can accelerate hypertension, diabetes, and metabolic syndrome, which are significant risk factors for cardiovascular diseases (CVDs) [2,3]. However, most studies have shown these associations in the advanced stages of CKD, including end-stage renal disease (ESRD) [4].

Early detection and management of CKD is a crucial medical issue before progression to ESRD, which is a progressive comorbidity that results in a range of complications, including anemia, malnutrition, bone, mineral metabolism disorder, and acidosis [5]. However, many people tend to overlook CKD at an earlier stage because they are often asymptomatic. In the Third National Health and Nutrition Survey (NHANES III), only 8% of patients were aware of their diseases among all subjects with a moderately decreased glomerular filtration rate (GFR) [6].

Hepatic steatosis is a significant public health problem worldwide because of its high incidence and progression to severe liver diseases, such as steatohepatitis, liver cirrhosis, and hepatocellular carcinoma [7]. The prevalence of liver steatosis is approximately 25% in adults worldwide [8], while epidemiological studies have shown that hepatic steatosis is closely related to obesity, dyslipidemia, and insulin resistance syndrome [9,10]. Moreover, hepatic steatosis is associated with extrahepatic complications, including atherosclerosis [11].

The prevalence of steatosis is higher in patients with CKD than in those without CKD. CKD is also more prevalent among subjects with hepatic steatosis than in those without because hepatic steatosis and CKD share common risk factors, such as obesity, hypertension, diabetes, dyslipidemia, metabolic syndrome, and atherosclerosis [12,13,14,15]. The incidence of simultaneous kidney-liver transplantation has increased gradually over the years [16]. Despite the potential connection between early CKD and steatosis on IHD, few studies have revealed a causal link between CKD and hepatic steatosis on cardiovascular risks due to similarity in traditional risk factors, such as hypertension, obesity, dyslipidemia, and insulin resistance. Accordingly, we prospectively investigated the combined effect of CKD and hepatic steatosis on incident IHD risk in large-scale, non-diabetic Koreans.

## 2. Materials and Methods

### 2.1. Study Population

This study was based on a population-based prospective cohort study of all participants in the Health Risk Assessment Study (HERAS). The aims and detailed information of the HERAS dataset were described in a previous study [17]. The cohort of adults aged more than 20 years in urban areas of South Korea were enrolled to explore surrogate indicators for cardiometabolic diseases by collecting the risk factors involved in the occurrence of cardiometabolic diseases and the lifestyle and health status of Koreans. A total of 20,530 adults aged 20–80 years were included in the baseline study conducted from 1 November 2006, to 8 June 2010. Subjects meeting any of the following criteria were excluded: previously diagnosed with angina pectoris, myocardial infarction, ischemic stroke, diabetes mellitus, or newly developed type 2 diabetes (defined as a fasting plasma glucose level ≥ 126 mg/dL), chronic kidney disease in advanced stage (G3b–5), alanine aminotransferase (ALT) ≥ 50 IU/L, aspartate aminotransferase (AST)/ALT > 2, positive for hepatitis B surface antigen or hepatitis C antibody, presence of liver cirrhosis, and any missing covariate information. The study protocol was approved by the institutional review board of the Yonsei University College of Medicine. Participants’ data were provided anonymously after they signed an informed consent form.

### 2.2. Anthropometric and Laboratory Measurements

Trained medical staff performed the physical examinations according to a standardized protocol. Bodyweight and height were measured to the nearest 0.1 kg and 0.1 cm, in light indoor clothing without footwear. Body mass index (BMI) was calculated as the ratio of body weight in kilograms to the square of height in meters (kg/m^2^). Systolic and diastolic blood pressures were measured on the patient’s right arm using a standard mercury sphygmomanometer in the sitting position after 10 min of rest (Baumanometer, W.A. Baum Co., Inc., Copiague, NY, USA). The mean arterial pressure was calculated from the systolic and diastolic blood pressures and were weighted as 1/3 systolic and 2/3 diastolic blood pressures. Data regarding health-related lifestyle behaviors, such as smoking, drinking, and physical activity, were obtained through structured questionnaires. Alcohol-related questions included the types of alcoholic drinks and the amount and frequency of alcohol consumption. Regular alcohol consumption was defined as alcohol consumption of ≥ 140 g of ethanol per week [18]. Regular exercise was defined as exercise more than three times per week [19]. Venous blood was sampled from each participant from an antecubital vein after more than 12-h fast. Fasting blood sugar, lipid profiles, AST, ALT, and creatinine levels were measured by enzymatic methods using a Hitachi 7600 automated chemistry analyzer (Hitachi Co., Tokyo, Japan). High-sensitivity C-reactive protein (hs-CRP) concentrations were measured with a Roche/Hitachi 912 System (Roche Diagnostics, Indianapolis, IN, USA) using a latex-enhanced immunoturbidimetric method.

### 2.3. Definition of CKD and Hepatic Steatosis

Estimated glomerular filtration rate (eGFR) was calculated using the abbreviated equation from the Modification of Diet in Renal Disease (MDRD). CKD was defined as the structural or functional kidney dysfunction, as determined by an eGFR value < 60 mL/min/1.73 m^2^ or proteinuria ≥ 1 + according to the Kidney Disease Outcomes Quality Initiative (KDOQI) CKD classification [20]. The diagnosis of hepatic steatosis was based on abdominal ultrasonography with a 3.5-Mhz transducer (HDI 5000, Philips, Bothell, USA). Ultrasonography was performed by the specialized radiologists. There was no information regarding this study. Patients were diagnosed with fatty liver if more than two of three findings were detected: deep attenuation, vascular blurring, and increased liver echogenicity [21].

### 2.4. Outcomes

The outcomes were acute myocardial infarction (ICD-10 code I21) or angina pectoris (ICD-10 code I20) after study enrollment. We conducted outcome assessments over the 50 months since the initial enrollment by linking each unique 13-digit identification number to the Health Insurance Review and Assessment (HIRA) database, which is derived from the universal coverage system in Korea.

### 2.5. Statistical Analysis

**Figure 1:**
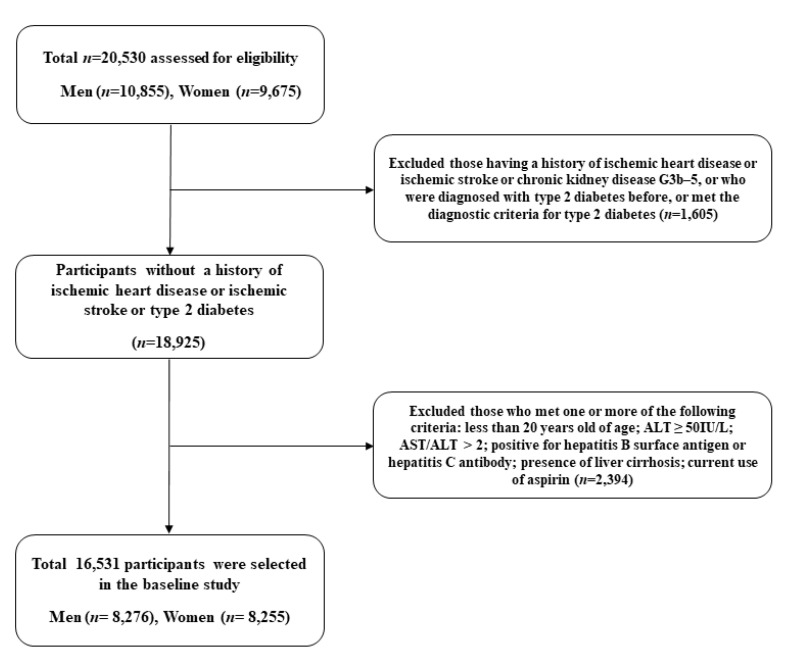
Flow chart of the study population selection.

All statistical analyses were performed using SAS software (version 9.4; SAS Institute Inc., Cary, NC, USA). Statistical significance was set at *p* < 0.05. To investigate the development of IHD, we excluded subjects with IHD and ischemic stroke at baseline. In addition, subjects with CKD (C3b-C5) and hepatic diseases, including alcoholic liver disease, viral hepatitis, and liver cirrhosis, were excluded. Furthermore, participants with type 2 diabetes were excluded, considering their potential effects on both CKD and IHD. Therefore, we excluded subjects with IHD, CKD in advanced stage, type 2 diabetes, and pre-existing hepatic disease at baseline. Finally, 16,531 subjects were eligible for the analysis (Figure 1). We divided the study population into four groups according to the existence of early CKD and hepatic steatosis: controls (group 1), early CKD only (group 2), hepatic steatosis only (group 3), and both early CKD and hepatic steatosis (group 4). The independent *t*-test for continuous variables was used to compare baseline characteristics of the study population with respect to new-onset IHD. The chi-square test categorical variables were used to evaluate the differences among the four groups. Continuous variables with normal distributions are presented as mean ± standard deviation (SD). Kaplan–Meier curves were used to assess the cumulative incidence of IHD. The log-rank test was used to determine whether the distribution of cumulative ischemic heart disease incidence differed between the groups. The hazard ratio (HR) and 95% confidence interval (CI) for IHD were calculated using Cox proportional hazards regression analyses after adjusting for potential confounding variables.

## 3. Results

A total of 16,531 subjects (8276 men and 8255 women) were enrolled in the final analysis. Table 1 shows the demographic and biochemical characteristics of the study participants in both groups. The mean of age, BMI, total cholesterol, triglycerides, high density-lipoprotein (HDL) cholesterol, and eGFR were 45.1 ± 10.5 years, 23.2 ± 3.0 kg/m^2^, 188.7 ± 33.3 mg/dL, 120.1 ± 81.8 mg/dL, 53.7 ± 12.7 mg/dL, and 83.7± 13.5, respectively. The mean values of mean arterial pressure, fasting plasma glucose, total cholesterol, triglyceride, and HDL cholesterol levels were highest in the group with both early CKD and hepatic steatosis (group 4). The prevalence of early CKD and hepatic steatosis was 5.8% and 30.1%, respectively.

Multivariate Cox regression analysis models were used to evaluate the relative contribution of CKD in earlier stages and hepatic steatosis to the development of IHD (Table 2). The presence of early CKD with hepatic steatosis was associated with a 76.0% increase in the risk of developing IHD compared with those without CKD and hepatic steatosis (HR = 1.76; 95% CI, 1.04–2.97) (model 3). However, the early CKD patients without hepatic steatosis showed no statistically significant difference in the risk of IHD development (HR = 1.26; 95% CI, 0.72–2.19). In addition, no significant difference was found in the risk of IHD when patients with hepatic steatosis were compared with the control group. 

**Figure 2:**
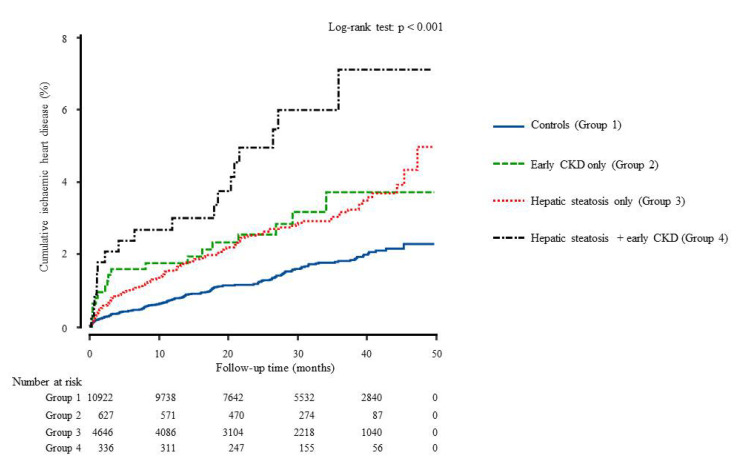
Cumulative incidence of ischemic heart disease during the follow-up time using Kaplan-Meier curve.

During the 50 months of follow-up, new-onset IHD developed in 326 individuals (2.0%, 326/16,531). Both early CKD and hepatic steatosis (group 4) showed the highest cumulative incidences of ischemic heart disease up to 50 months after the baseline survey (log-rank test, *p* < 0.001) (Figure 2). The incidence rates per 1000 person-years were 6.3, 12.6, 11.8, and 23.3 in the four groups. Furthermore, to assess the features of the combined effect, we compared the HR according to the presence of each individual and both together among all participants. Table 3 shows that the joint effect of early CKD and hepatic steatosis has synergistic effects on the development of IHD.

## 4. Discussion

In this large cohort study, early CKD with hepatic steatosis preceded and predicted new-onset IHD among individuals without diabetes. These relationships persisted after adjusting for lifestyle factors, inflammatory markers, and metabolic risk factors.

We found a combined effect of early CKD and liver steatosis on the incidence of IHD. Previous studies have shown that CKD is independently associated with coronary artery disease [22,23]. The severity of liver steatosis is positively related to the degree of coronary atherosclerosis [24]. However, the combined effect of early CKD and steatosis on IHD has not been thoroughly examined in a longitudinal study. Our results showed that early CKD with hepatic steatosis was significantly associated with the incidence of IHD, even though early CKD and hepatic steatosis were not significantly related to the incidence of IHD. This implies that early CKD can be mechanistically linked to hepatic steatosis.

Although it is challenging to uncover a causal relationship between liver steatosis and CKD, we can find putative mechanisms by understanding the confounding factors linking hepatic steatosis and CKD at an earlier stage. The potential pathogenesis contributing to CKD and steatosis could be nutritional factors, such as fructose and vitamin D. Fructose is the main constituent of sugar sweeteners. It may lead to kidney and liver injury through a mechanism mediated by uric acid overproduction. Fructokinase (KRK) is the first enzyme that metabolizes fructose. The metabolism of fructose to fructose-1-phosphate by KRK occurs primarily in the liver without any negative feedback. Its metabolism requires dephosphorylation of ATP with AMP and a fall in intracellular phosphate. Phosphate stimulates AMP deaminase (AMPD), catalyzes the degradation of AMP to inosine monophosphate, and finally produces uric acid. The uric acid released from the liver circulates to the kidney, thereby damaging the kidney [25]. Furthermore, previous studies have shown that uric acid-lowering agents have improved fructose-induced CKD and steatosis [26].

Vitamin D plays many roles in the body, such as immunity, inflammation, regulation of cell proliferation, and metabolism. Vitamin D deficiency is associated with the pathogenesis of CKD and hepatic steatosis [27,28]. It is produced on the skin through a UV-mediated reaction and is metabolized to its active form (1α, 25 (OH)_2_) through hydroxylation exerted by the kidney and liver, respectively. Vitamin D regulates the metabolism of free fatty acids (FFAs) through its action on peroxisome proliferator-activated receptor gamma (PPAR-γ), improving FFA-induced insulin resistance in vitro [29]. Accordingly, vitamin D deficiency leads to increased FFA levels. Moreover, the increased FFAs circulate in the blood, induce fat accumulation in the liver, and promote the development of CKD and steatosis.

Cellular metabolism is orchestrated by the molecular sensors of energy, oxygen, and nutrients. The dysfunction of some of these sensors, including fetuin-A, adiponectin and 5′-AMP-activated protein kinase (AMPK), has been involved in the pathogenesis of CKD and hepatic steatosis. Fetuin-A is an important promoter of insulin resistance. It is produced by the liver and is secreted into the bloodstream. It binds and inhibits the insulin receptor tyrosine kinase, leading to insulin resistance. In humans, higher fetuin-A levels are associated with insulin resistance in patients with CKD [30]. In contrast to fetuin-A, adiponectin improves insulin sensitivity. Serum fetuin-A and adiponectin levels are inversely correlated with each other [31]. In the kidney and liver, AMPK is critical for directing renal podocytes and hepatocytes to detrimental pathways, resulting in end-organ damage by inflammatory and fibrotic cascades [31]. Consequently, CKD and hepatic steatosis share a common proinflammatory pathogenesis in disease progression [32,33].

Despite the large cohort dataset, there were several limitations to our study. First, the study population may not be representative of the general Korean population. Subjects who participate in health checkups are usually interested in their health problems, leading to selection bias. Second, the gold standard for the diagnosis of hepatic steatosis is liver biopsy, but we used ultrasonographic evaluations to define hepatic steatosis because it has limited availability in health examination settings. Third, this study did not reflect the effect of changes in the severity of hepatic steatosis or CKD stage, which could affect the incidence of IHD during the follow-up period. Lastly, because we did not consider the type of hypertension medicine, these variables were not fully adjusted for in the statistical models.

## 5. Conclusions

In conclusion, even less than stage 3A CKD could predict IHD in patients with hepatic steatosis. Timely detection of both CKD and steatosis, even in the earlier stages, is critical to prevent the development of IHD. Further studies are needed to delineate the direct relationship between liver steatosis and CKD.

## Figures and Tables

**Table 1:** Baseline characteristics of the study population.

	Controls (*n* = 10,922)	Early CKD Only (*n* = 627)	Hepatic Steatosis Only (*n* = 4646)	Hepatic Steatosis + Early CKD (*n* = 336)	*P* value ^1^	Post Hoc ^2^
Age (years)	44.0 ± 10.5	46.4 ± 12.3	47.1 ± 9.9	50.7 ± 11.0	<0.001	a,b,c,e,f
Male sex (%)	41.6	49.6	68.5	71.7	<0.001	-
BMI (kg/m^2^)	22.3 ± 2.6	22.3 ± 2.7	25.3 ± 2.7	25.0 ± 2.5	<0.001	b,c,d,e
Systolic BP (mmHg)	118.8 ± 14.9	121.5 ± 16.0	127.1 ± 14.5	130.3 ± 15.9	<0.001	a,b,c,d,e,f
Diastolic BP (mmHg)	74.0 ± 9.7	75.9 ± 10.2	79.6 ± 9.6	81.9 ± 9.9	<0.001	a,b,c,d,e,f
AST (IU/L)	19.2 ± 5.0	20.7 ± 5.7	21.6 ± 5.9	23.1 ± 5.9	<0.001	a,b,c,d,e,f
ALT (IU/L)	17.4 ± 7.5	19.1 ± 7.9	24.8 ± 9.5	26.4 ± 9.3	<0.001	a,b,c,d,e,f
GGT (IU/L)	23.9 ± 22.9	30.1 ± 31.7	37.3 ± 31.3	40.3 ± 32.6	<0.001	a,b,c,d,e
FPG (mg/dL)	89.3 ± 8.9	90.7 ± 10.6	95.0 ± 10.0	96.3 ± 10.9	<0.001	a,b,c,d,e
Total cholesterol (mg/dL)	183.9 ± 31.8	191.1 ± 35.0	198.5 ± 34.1	205.1 ± 35.0	<0.001	a,b,c,d,e,f
Triglyceride (mg/dL)	101.0 ± 56.4	105.3 ± 59.7	162.7 ± 110.2	176.7 ± 105.4	<0.001	b,c,d,e,f
HDL-C (mg/dL)	56.3 ± 12.6	56.8 ± 14.6	47.8 ± 10.2	48.0 ± 11.1	<0.001	b,c,d,e
Hs-CRP (mg/L)	1.1 ± 2.8	1.9 ± 5.2	1.7 ± 4.0	2.1 ± 6.0	<0.001	a,b,c
Current smoker (%)	21.2	27.8	30.8	26.8	<0.001	-
Alcohol drinking (%)	41.5	45.4	47.5	45.1	<0.001	-
Regular exercise (%)	31.8	32.5	29.4	32.5	0.022	-
Hypertension (%)	14.3	24.2	30.1	41.4	<0.001	-
Hepatic steatosis severity ^3^	0.0 ± 0.0	0.0 ± 0.0	1.8 ± 1.2	1.9 ± 1.2	<0.001	b,c,d,e,f
eGFR	85.0 ± 13.3	74.7 ± 16.4	82.7 ± 12.2	71.4 ± 14.5	<0.001	a,b,c,d,e,f

^1^ *p*-values were calculated using one-way ANOVA or Pearson’s chi-square test. ^2^ Post hoc analysis with the Bonferroni method: a, Q1 versus Q2; b, Q1 versus Q3; c, Q1 versus Q4; d, Q2 versus Q3; e, Q2 versus Q4; and f, Q3 versus Q4. **^3^** Average grade of hepatic steatosis via ultrasound.

**Table 2:** Hazard ratios and 95% confidence intervals for new-onset ischemic heart diseases.

		Controls (*n* = 10,922)	Early CKD Only (*n* = 627)	Hepatic Steatosis Only (*n* = 4646)	Hepatic Steatosis + Early CKD (*n* = 336)
New cases of ischemic heart disease, *n*		164	18	126	18
Mean follow-up, years		2.4 ± 1.1	2.3 ± 0.9	2.3 ± 1.1	2.3 ± 1.0
Pearson-years of follow-up		26,023	1427	10,636	773
Incidence rate/1000 person–years		6.3	12.6	11.8	23.3
Model 1	HR (95% CI)	1.00 (reference)	1.51 (0.93–2.47)	1.47 (1.16–1.86)	2.16 (1.32–3.53)
	*p* value	-	0.097	0.001	0.002
Model 2	HR (95% CI)	1.00 (reference)	1.34 (0.77–2.32)	1.29 (0.98–1.69)	1.95 (1.16–3.27)
	*p* value	-	0.299	0.068	0.011
Model 3	HR (95% CI)	1.00 (reference)	1.26 (0.72–2.19)	1.19 (0.90–1.57)	1.76 (1.04–2.97)
	*p* value	-	0.425	0.230	0.033

Model 1: adjusted for age and sex. Model 2: adjusted for age, sex, body mass index, smoking status, alcohol intake, and physical activity. Model 3: adjusted for age, sex, body mass index, smoking status, alcohol intake, physical activity, mean arterial blood pressure, fasting plasma glucose, high density-lipoprotein cholesterol, γ-glutamyltransferase, high-sensitivity C-reactive protein level, and hypertension medication.

**Table 3:** Multivariate Cox proportional-hazards regression models for incident ischemic heart disease by early CKD only, hepatic steatosis only, or the presence of both.

	Early CKD Only		Hepatic Steatosis Only		The Presence of Both	
	HRs (95% CIs)	*p* value	HRs (95% CIs)	*p* value	HRs (95% CIs)	*p* value
Age, years	1.06 (1.05–1.07)	<0.001	1.06 (1.05–1.07)	<0.001	1.06 (1.05–1.07)	<0.001
Male sex, yes vs. no	1.34 (0.94–1.90)	0.105	1.34 (0.94–1.91)	0.106	1.34 (0.94–1.90)	0.106
Body mass index, kg/m^2^	1.04 (0.99–1.09)	0.094	1.03 (0.98–1.08)	0.276	1.03 (0.98–1.08)	0.249
Current smoking, yes vs. no	1.22 (0.84–1.77)	0.297	1.21 (0.84–1.76)	0.307	1.22 (0.84–1.77)	0.300
Alcohol drinking, yes vs. no	0.77 (0.59–0.99)	0.047	0.77 (0.59–1.00)	0.050	0.77 (0.59–1.00)	0.052
Regular exercise, yes vs. no	1.18 (0.93–1.50)	0.170	1.19 (0.94–1.51)	0.156	1.19 (0.94–1.51)	0.158
Mean arterial pressure, mmHg	0.99 (0.98–1.01)	0.334	0.99 (0.98–1.01)	0.319	0.99 (0.98–1.01)	0.298
Fasting plasma glucose, mg/dL	1.02 (1.00–1.03)	0.011	1.02 (1.00–1.03)	0.017	1.01 (1.00–1.03)	0.020
HDL-cholesterol, mg/dL	0.99 (0.98–1.00)	0.118	0.99 (0.98–1.00)	0.183	0.99 (0.98–1.00)	0.187
γ-glutamyltransferase, IU	1.00 (1.00–1.01)	0.733	1.00 (1.00–1.01)	0.797	1.00 (1.00–1.01)	0.820
High-sensitivity C-reactive protein, mg/L	1.00 (0.96–1.03)	0.748	1.00 (0.97–1.03)	0.779	1.00 (0.96–1.03)	0.752
Hypertension medication, yes vs. no	1.71 (1.26–2.32)	<0.001	1.73 (1.27–2.34)	<0.001	1.71 (1.26–2.32)	<0.001
Early CKD, yes vs. no	1.38 (0.95–2.02)	0.093	-	-	-	-
Hepatic steatosis, yes vs. no	-	-	1.22 (0.93–1.59)	0.150	-	-
The presence of both, yes vs. controls	-	-	-	-	1.76 (1.04–2.97)	0.033

## Data Availability

The data underlying this article will be shared upon reasonable request from the corresponding author.
